# An integrated approach to groundwater potential–vulnerability mapping using AHP and DRASTIC

**DOI:** 10.1038/s41598-026-49788-2

**Published:** 2026-04-24

**Authors:** Bahman Fazil Fatih, Asghar Asghari Moghaddam, Twana O. Abdullah, Nadhir Al-Ansari

**Affiliations:** 1 Department of Earth Sciences, Faculty of Natural Sciences, University of Tabriz, Tabriz, Iran; 2 Groundwater Directorate of Sulaimani, Sulaymaniyah, Kurdistan Region Iraq; 3 Luleå University of Technology, Luleå, 971 87 Sweden

**Keywords:** Groundwater potential, Groundwater vulnerability, Potential–vulnerability (PV) integration, AHP, DRASTIC, ROC analysis, Semi-arid region, Environmental sciences, Hydrology

## Abstract

Groundwater in semi-arid regions is increasingly stressed by intensive abstraction and contamination, while aquifer productivity and intrinsic vulnerability are commonly evaluated separately. This study investigates whether structured integration of groundwater potential and intrinsic vulnerability can provide a more reliable basis for sustainable groundwater management in the Halabja–Khwrmal area, northeast Iraq. Groundwater potential was delineated using the Analytical Hierarchy Process (AHP) applied to seven hydrogeological and environmental factors, whereas intrinsic vulnerability was assessed using the standard DRASTIC model. Both indices were independently validated prior to integration. Receiver Operating Characteristic (ROC) analysis based on 430 well discharge records yielded an Area Under the Curve (AUC) of 0.751, indicating acceptable discrimination of productive zones. Regression between DRASTIC index values and measured nitrate concentrations showed a strong positive relationship (R² = 0.797), supporting vulnerability reliability. Cross-classification of the validated ordinal groundwater potential and vulnerability indices generated nine Potential–Vulnerability zones, where highly productive areas largely coincide with low intrinsic vulnerability. High groundwater potential occupies 49.24% of the basin, while high intrinsic vulnerability covers 20.65%, with limited spatial overlap between highly productive and highly vulnerable conditions. The class-preserving integration prevents compensatory masking between productivity and susceptibility and provides a transparent spatial framework for regulated abstraction and priority protection. The proposed Potential–Vulnerability framework offers a transferable spatial basis for groundwater management in hydrogeologically variable semi-arid aquifer systems.

## Introduction

Groundwater represents the principal freshwater reserve in semi-arid regions, where surface water availability is spatially limited and seasonally variable^1^. In northeast Iraq, particularly within the Halabja–Khwrmal area (HKA), groundwater sustains domestic supply, irrigation, and local economic activity^2^. Progressive abstraction, agricultural intensification, and land-use transformation have modified recharge conditions and increased pressure on aquifer systems in the area. Comparable semi-arid agricultural basins have exhibited similar responses, including nitrate enrichment and salinization associated with intensive land management^3^. In such hydro-environmental settings, groundwater management requires spatial discrimination of zones characterized by favorable productivity while simultaneously maintaining intrinsic protection against contamination^4^.

Groundwater potential mapping (GWPM) and groundwater vulnerability mapping (GWVM) constitute two established analytical directions. GWPM delineates areas of relative productivity through integration of hydrogeological and environmental controls^5,6^. GWVM evaluates the inherent sensitivity of aquifers to surface-derived pollutants, commonly through index-based frameworks such as DRASTIC^7^. In semi-arid basins, recent refinements have incorporated modified recharge representation and alternative weighting strategies to improve vulnerability discrimination^8^. The systematic comparison of established methods and subsequent sensitivity analysis has further enhanced interpretation of contamination susceptibility under agricultural stress conditions^9^. The integration of hazard quantification with vulnerability assessment has enabled more comprehensive risk characterization in intensively cultivated basins^10^.

Despite methodological maturity, groundwater potential and intrinsic vulnerability are frequently evaluated independently. Although vulnerability has been refined through incorporation of land use and anthropogenic pressure within modified index-based approaches in semi-arid basin studies^11^, aquifer productivity has rarely been incorporated within the same structured decision framework while preserving categorical integrity. A spatial approach that maintains the independent class identity of both indices and validates each prior to integration remains comparatively limited, particularly in structurally complex mountainous terrains.

The Halabja–Khwrmal area exhibits heterogeneous lithostratigraphy, fractured carbonate and clastic units, variable slope gradients, and spatially uneven recharge regimes. Groundwater dependence is high, and both abstraction pressure and contamination risk display marked spatial variability. To date, no integrated Potential–Vulnerability (PV) assessment for the HKA has independently validated groundwater productivity and intrinsic vulnerability prior to structured integration.

To clarify the specific contribution of this study, the proposed framework does not adopt a conventional additive overlay of groundwater potential and vulnerability indices, nor does it apply normalization procedures that rescale both models into a common continuous range prior to integration. Groundwater productivity and intrinsic vulnerability are evaluated independently within their respective conceptual structures and are each subjected to empirical validation using field-based observations, including well-yield data for potential assessment and measured nitrate concentrations for vulnerability appraisal. Integration is performed only after independent validation, through a structured cross-classification scheme that preserves ordinal class identity and avoids numerical amplification. Within this configuration, productivity and susceptibility remain analytically independent, preventing compensatory effects between high yield capacity and elevated vulnerability.

Accordingly, this study aims to (i) delineate groundwater potential using an AHP-based multi-criteria approach and evaluate its predictive performance against observed well-yield data, (ii) assess intrinsic vulnerability using the standard DRASTIC model and examine its correspondence with measured nitrate concentrations, and (iii) integrate both validated indices through a structured cross-classification scheme that maintains categorical integrity. The resulting Potential–Vulnerability framework provides a spatially explicit basis for distinguishing areas suitable for regulated groundwater development from zones requiring priority protection within a semi-arid hydrogeologically variable system.

## Materials and methods

### Study area

**Fig. 1:**
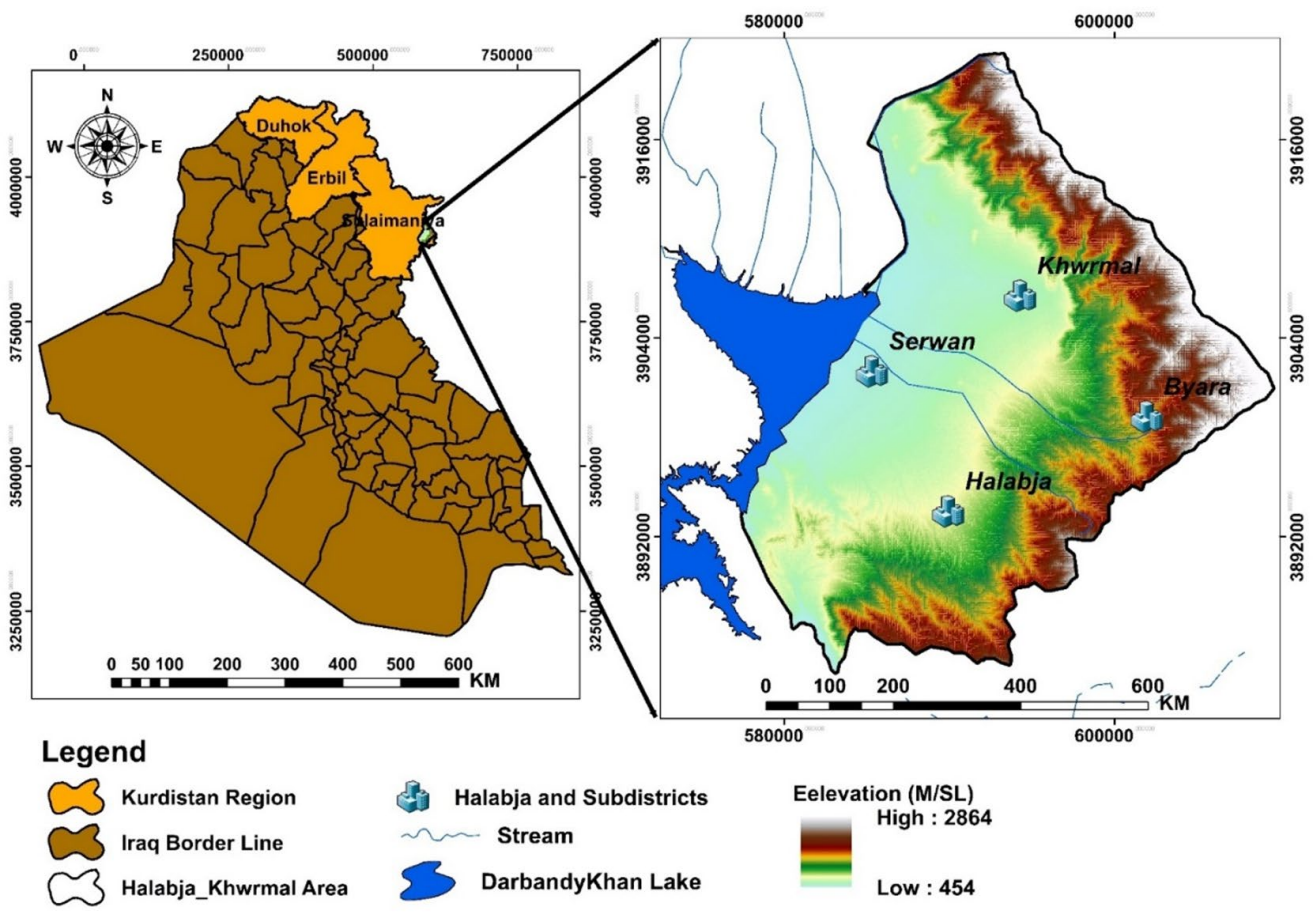
Location map of the study area (This figure was prepared using ArcGIS Desktop 10.8 (https://www.esri.com/en-us/arcgis/products/arcgis-desktop/overview)).

The study area is located in northeastern Iraq near the Iranian border within UTM Zone 38 N, extending from 577,486 to 609,531 m (E) and 3,838,848–3,921,133 m (N) (Fig. 1). The basin covers nearly 615 km², with elevations ranging from about 454 m in the central lowlands to 2,864 m in surrounding high-relief zones, generating strong spatial contrasts in runoff and recharge. The population is estimated at approximately 120,000 inhabitants^12^.

The climate is semi-arid with Mediterranean influence, characterized by wet winters and dry summers^13^. Annual precipitation ranges from 522 to 771 mm and is concentrated between November and April^14^, while temperatures vary from about 3.3 °C in winter to 43.4 °C in summer^15^. The seasonal concentration of rainfall, combined with high summer evapotranspiration, exerts strong control on recharge dynamics.

Geologically, the basin forms part of the Zagros fold–thrust belt and comprises limestone, dolomitic limestone, sandstone, conglomerate, and Quaternary alluvial deposits^16^. Folded and faulted carbonate and clastic formations dominate the structural framework^17,18^, and thrust-related fracture systems influence groundwater storage and flow^19,20^.

Hydrogeologically, karstic and fissured-carbonate aquifers coexist with intergranular alluvial units and low-permeability aquitards^21^. The alluvial aquifer occupies approximately 53% of the basin and represents the most productive unit. Groundwater levels are shallow in the central plains and deepen toward upland areas, with regional flow predominantly directed westward toward Darbandykhan Lake^22^.

### Data sources and criteria selection

Groundwater potential and intrinsic vulnerability were evaluated using two groups of thematic factors representing the principal hydrogeological controls on recharge, storage, transmissivity, and contamination susceptibility within the basin. Factor selection was guided by regional geological conditions and established GIS-based assessment approaches, combining multi-criteria decision analysis for groundwater potential evaluation^23^, with the index-based DRASTIC framework for vulnerability assessment^11^. Both models were developed and validated independently prior to integration in order to preserve the ordinal significance of each index and avoid bias associated with direct additive or normalization procedures.

A total of fourteen spatial layers were prepared, comprising seven hydrogeological and environmental factors for groundwater potential mapping (GWPM) and seven standard DRASTIC parameters for groundwater vulnerability mapping (GWVM). Validation employed observed well-yield data for the potential model and measured nitrate (NO₃⁻) concentrations for the vulnerability model.

All datasets were projected to UTM Zone 38 N and processed in ArcGIS 10.8. Raster layers were standardized to a uniform 30 m spatial resolution and clipped to the study area boundary prior to classification according to model-specific rating schemes. The thematic datasets, sources, and processing procedures used for model development are summarized in Table 1, while the independent validation datasets are presented in Table 2.

**Table 1:** Data sources and thematic layers employed in groundwater potential and vulnerability assessment.

No.	Layer/Parameter	Data Source(s)	Processing
Groundwater Potential Mapping (GWPM)			
1	Rainfall	Meteorological station records	IDW interpolation
2	Hydrogeological Units	*GD Sulaimani & relevant published literature	Digitized and aquifer-classified
3	Land Use/Land Cover (LULC)	Landsat satellite imagery (USGS Earth Explorer)	Supervised classification (Random Forest)
4	Slope	SRTM DEM (30 m), (USGS)	DEM-derived slope
5	Soil Type	FAO^24^ & Berding^25^	Permeability-based reclassification
6	Lineament Density	DEM and Landsat imagery	Extracted and density mapped
7	Drainage Density	SRTM DEM (USGS Earth Explorer)	Stream extraction and density mapping
Groundwater Vulnerability Mapping (GWVM – DRASTIC)			
8	Depth to Water Table (D)	Borehole data, GD Sulaimani	Interpolated from borehole data
9	Net Recharge (R)	Meteorological stations & hydrogeological studies	Rainfall-based estimation
10	Aquifer Media (A)	Geology & borehole data; GD Sulaimani; Iraq Geological Survey	Lithological classification
11	Soil Media (S)	FAO^24^ & Berding^25^	Texture-based classification
12	Topography (T)	Digital elevation data (DEM), USGS Earth Explorer	Slope-derived (%)
13	Vadose Zone (I)	Lithology; GD Sulaimani	Unsaturated zone classification
14	Hydraulic Conductivity (C)	Well tests; GD Sulaimani & published literature	Pumping-test derived and interpolated

*GD Sulaimani: Groundwater Directorate of Sulaimani^26^.

**Table 2:** Independent datasets used for validation of groundwater potential and vulnerability.

No.	Layer/Parameter	Data Source(s)	Processing
Groundwater Potential Mapping (GWPM)			
1	Rainfall	Meteorological station records	IDW interpolation
2	Hydrogeological Units	*GD Sulaimani & relevant published literature	Digitized and aquifer-classified
3	Land Use/Land Cover (LULC)	Landsat satellite imagery (USGS Earth Explorer)	Supervised classification (Random Forest)
4	Slope	SRTM DEM (30 m), (USGS)	DEM-derived slope
5	Soil Type	FAO^24^ & Berding^25^	Permeability-based reclassification
6	Lineament Density	DEM and Landsat imagery	Extracted and density mapped
7	Drainage Density	SRTM DEM (USGS Earth Explorer)	Stream extraction and density mapping
Groundwater Vulnerability Mapping (GWVM – DRASTIC)			
8	Depth to Water Table (D)	Borehole data, GD Sulaimani	Interpolated from borehole data
9	Net Recharge (R)	Meteorological stations & hydrogeological studies	Rainfall-based estimation
10	Aquifer Media (A)	Geology & borehole data; GD Sulaimani; Iraq Geological Survey	Lithological classification
11	Soil Media (S)	FAO^24^ & Berding^25^	Texture-based classification
12	Topography (T)	Digital elevation data (DEM), USGS Earth Explorer	Slope-derived (%)
13	Vadose Zone (I)	Lithology; GD Sulaimani	Unsaturated zone classification
14	Hydraulic Conductivity (C)	Well tests; GD Sulaimani & published literature	Pumping-test derived and interpolated

*GD Sulaimani: Groundwater Directorate of Sulaimani^26^.

### Methodological framework

The methodological workflow comprised four stages: (1) preparation of thematic layers, (2) groundwater potential modeling using the Analytical Hierarchy Process (AHP), (3) intrinsic vulnerability assessment using the standard DRASTIC model, and (4) structured integration of the validated outputs into a Potential–Vulnerability (PV) framework.

For groundwater potential mapping, thematic layers were standardized to a uniform 30 m spatial resolution, reclassified, and combined using AHP-derived weights through weighted overlay analysis. Vulnerability was evaluated by rating and weighting the seven DRASTIC parameters to compute the composite index.

**Fig. 2:**
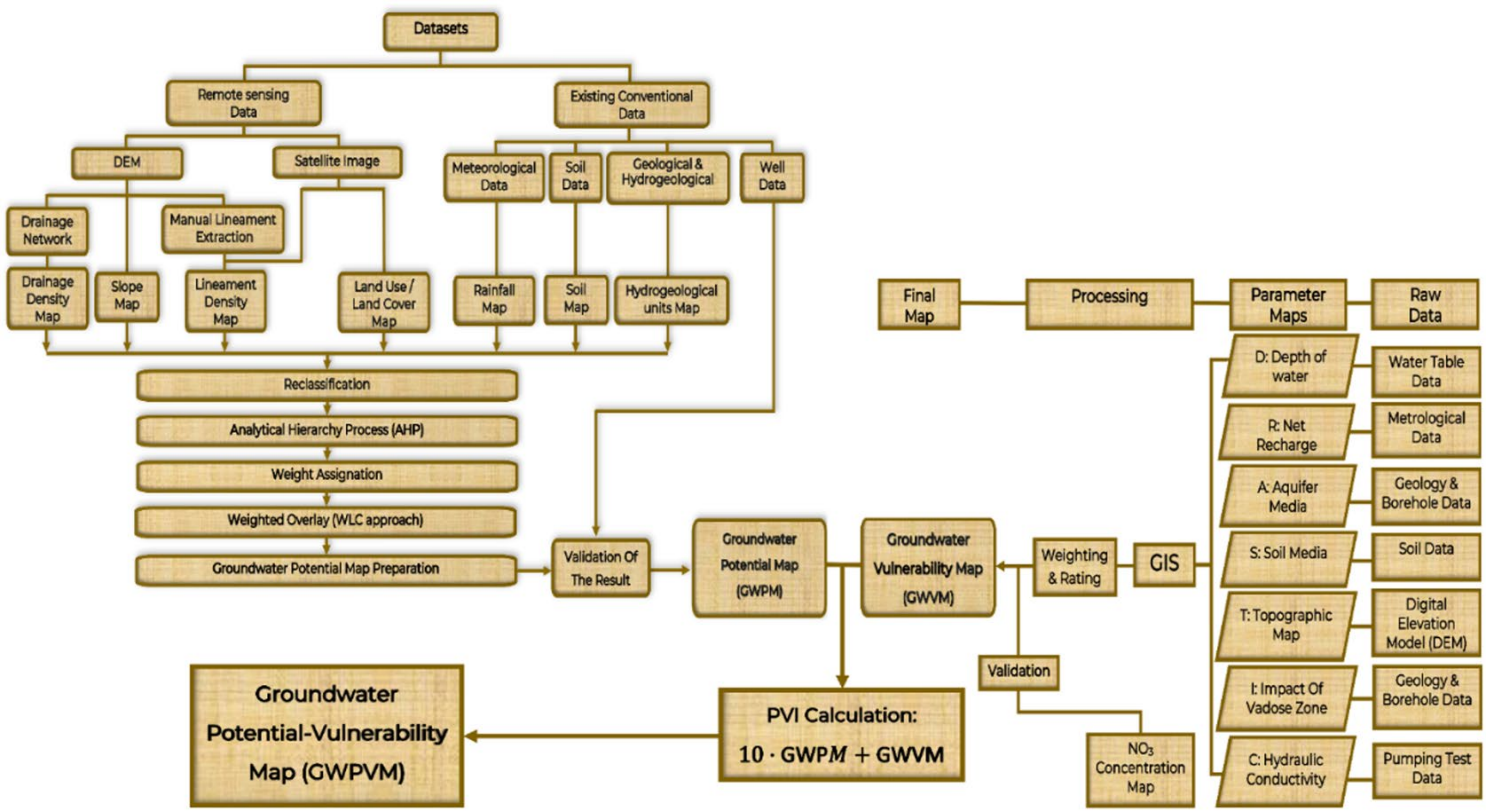
Methodological workflow for groundwater potential (AHP) and vulnerability (DRASTIC) assessment with independent validation and cross-classification.

Both indices were independently validated prior to integration. Groundwater potential was assessed using well discharge data through binary ROC–AUC analysis, whereas nitrate (NO₃⁻) concentrations were used to evaluate vulnerability performance. The validated indices were reclassified into three ordinal categories (1 = low, 2 = moderate, 3 = high) and integrated through a cross-classification matrix without normalization or rescaling, generating nine PV zones. The workflow is illustrated in Fig. 2.

### Groundwater potential modeling using AHP

Groundwater potential was assessed in a GIS environment using a Weighted Linear Combination (WLC) method^27^. Factor weights were derived through the Analytical Hierarchy Process (AHP)^28^. This approach has been applied widely in semi-arid regions where groundwater occurrence depends on spatially variable hydrogeological conditions^23,29^.

Seven factors were used: rainfall, hydrogeological unit, lineament density, slope, soil type, land use/land cover (LULC), and drainage density. In the carbonate–alluvial aquifer system, these variables describe recharge input, permeability contrasts, structural control of flow, and terrain-related redistribution processes^30^.

Weights were assigned according to dominant physical controls on groundwater occurrence. Rainfall (22%) received the highest weight, reflecting the dependence of recharge on seasonal precipitation in semi-arid fractured aquifer settings^31^. Hydrogeological units (20%) represent variations in storage and hydraulic conductivity, especially within fractured formations^32^. Lineament density (16%) accounts for fracture-enhanced secondary porosity and permeability, which facilitates groundwater flow^33^. Land use/land cover (14%), soil type (11%), slope (9%), and drainage density (8%) influence infiltration and runoff behaviors. Consistent with previous studies in comparable geological environments, recharge potential and lithological permeability were interpreted as exerting stronger control on groundwater occurrence than surface redistribution processes^34^.

Pairwise comparisons followed Saaty’s 1–9 fundamental scale of relative importance. Consistency was checked using the Consistency Index (CI) and Consistency Ratio (CR), with CR < 0.10 accepted as indicating reliable judgments^35^. Final weights and suitability ratings (1–5) are shown in Table 3.

**Table 3:** Factor weights derived through AHP for groundwater potential mapping.

No.	Criteria	Classes	Rank	Factor Weight (%)
1.	Rainfall (mm/year)	> 700	5	22
		650.01–700	4	
		600.01–650.0	3	
		550.01–600.0	2	
		≤ 550	1	
2.	Hydrogeological Unit	Karstic Aquifer	5	20
		Karstic Fissured Aquifer	4	
		Intergranular Aquifer	3	
		Aquitard	1	
3.	Lineament Density (km/km²)	> 2.0	5	16
		1.501–2.0	4	
		1.01–1.50	3	
		0.501–1.0	2	
		≤ 0.5	1	
4.	Slope (degrees)	< 10.0	5	9
		10.01–25.0	4	
		25.01–40.0	3	
		40.01–65.0	2	
		> 65	1	
5.	Soil Type	Gravel/Absent (B2)	5	11
		Silty Loam (C3)	3	
		Clay (C2.1)	1	
6.	Land Use/Land Cover (LULC)	Vegetation	5	14
		Agriculture	4	
		Water	3	
		Bareland	2	
		Settlements	1	
7.	Drainage Density (km/km²)	< 0.45	5	8
		0.451–0.90	4	
		0.901–1.90	3	
		1.901–2.0	2	
		> 2.0	1	

**Fig. 3:**
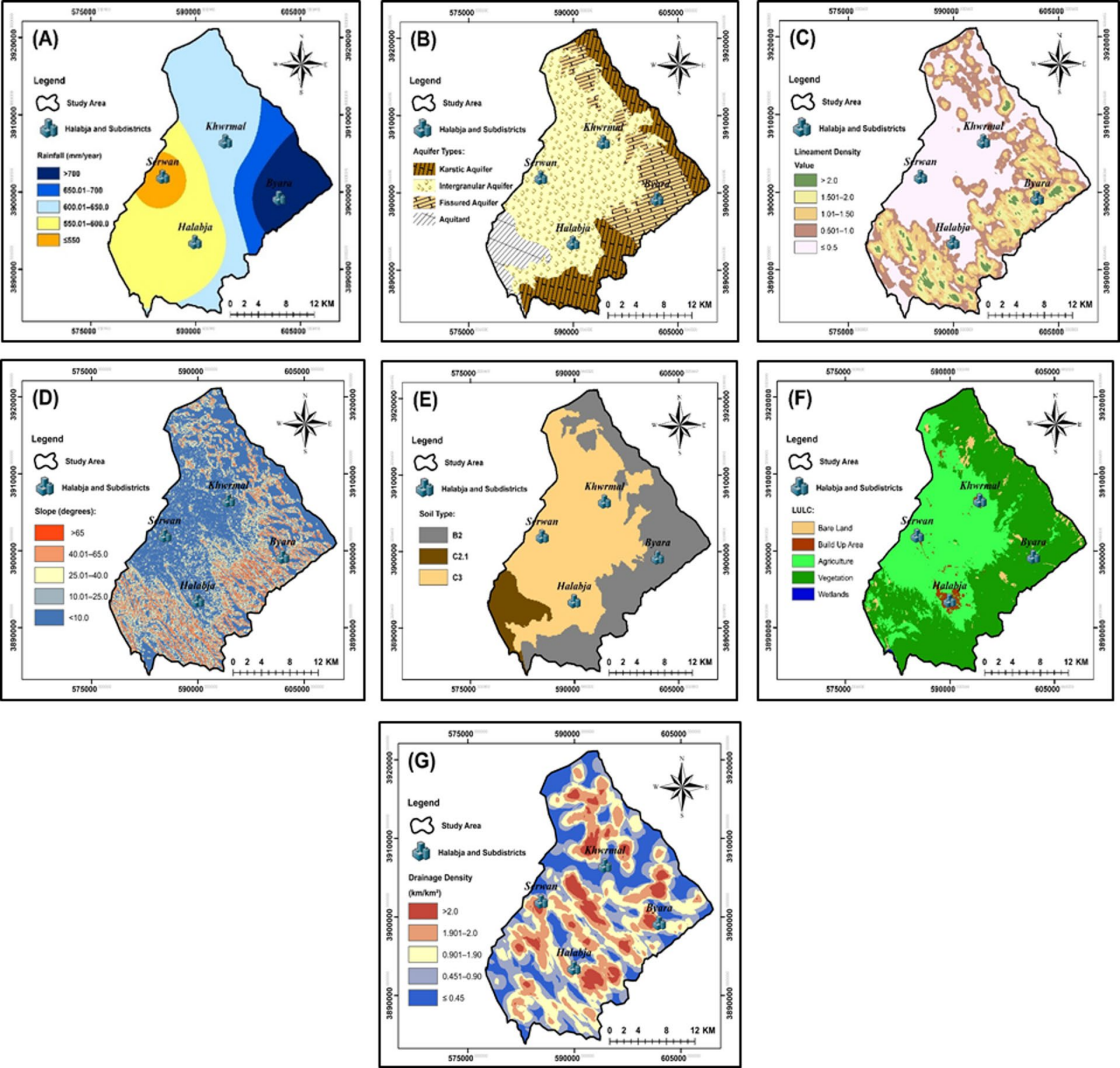
Input layers for groundwater potential mapping: **(a)** Rainfall distribution, **(b)** Hydrogeological units, **(c)** Lineament density **(d)** Slope, **(e)** Soil cover, **(f)** Land use/land cover, and **(g)** Drainage density. (This figure was prepared using ArcGIS Desktop 10.8 (https://www.esri.com/en-us/arcgis/products/arcgis-desktop/overview)).

The Groundwater Potential Index (GWPI) was calculated as (Eq. 1):

1 $$\:GWPI=\sum\:({W}_{i}\times\:{R}_{i})$$

where $\:{W}_{i}$ denotes the AHP-derived weight of factor *i* and $\:{R}_{i}$ the corresponding suitability rating. The resulting index was classified into low, moderate, and high groundwater potential zones. The spatial distribution of input layers is shown in Fig. 3 (a–g).

### Groundwater vulnerability assessment using the DRASTIC model

Intrinsic vulnerability was evaluated using the standard DRASTIC index^7^, which incorporates seven hydrogeological parameters controlling contaminant migration: depth to water (D), net recharge (R), aquifer media (A), soil media (S), topography (T), impact of the vadose zone (I), and hydraulic conductivity (C). The framework reflects inherent system sensitivity rather than contamination linked to specific anthropogenic activities and has been implemented under comparable climatic and geological settings^11^.

Depth to water (weight = 5) was represented by groundwater level depth, where shallow conditions were assigned higher ratings in accordance with the DRASTIC framework. Net recharge (weight = 4) was treated as the volume of infiltrating water available for downward transport of dissolved constituents. Aquifer media (weight = 3) was interpreted through its lithological influence on storage and permeability, with fractured carbonate formations and coarse-grained deposits receiving higher ratings. Soil media (weight = 2) was considered in relation to near-surface infiltration characteristics. Topography (weight = 1), derived from slope, was associated with runoff tendencies and surface residence time. The vadose zone parameter (weight = 5) reflected the role of unsaturated lithology in subsurface contaminant transfer, while hydraulic conductivity (weight = 3) corresponded to the capacity for lateral movement within the aquifer system. Parameter ranges and ratings were assigned in accordance with the original DRASTIC scheme (Table 4).

**Table 4:** DRASTIC parameter weights and rating intervals following the original scheme.

D: Depth to Water (Weight:5)		*R*: Net Recharge (Weight:4)		A: Aquifer Media (Weight:3)		S: Soil Media (Weight:2)	
Range (m)	Rating	Range (mm/year)	Rating	Type(s)	Rating	Type(s)	Rating
> 100	1	< 50	1	Metamorphic/Igneous rocks	3	Silty loam	4
90.01–100	2	50.01–100	3	Bedded sandstone, limestone, & shale	6	Shrinking or aggregated clay	7
75.01–90.0.01.0	3	100.01–175	6	Massive sandstone	6	Thin or absent, gravel	10
60.01–75.0.01.0	4	175.01–250	8	Massive limestone	8		
45.01–60.0.01.0	5	> 250	9	Sand and Gravel	8		
30.01–45.0.01.0	6			Karst limestone	10		
20.01–30.0.01.0	7						
10.01–20.0.01.0	8						
5.01–10.0.01.0	9						
0–5.0	10						

T: Topography (Weight:1)		I: Vadose Zone Impact (Weight:5)				C: Hydraulic conductivity (Weight:3)	
(slope %)	Rating	Type(s)			Rating	Range (m/day)	Rating
> 18	1	Sand & gravel with silt/clay (less permeable)			5	< 4.0	1
12.01–18.01	3	Sand & Gravel (moderately permeable)			8	4.01–12.0.01.0	2
6.01–12.0.01.0	5	Karst limestone			10	12.01–30.0.01.0	4
2.01–6.0.01.0	9					30.01–40.0.01.0	6
0–2.0	10					40.01–80.01	8
						> 80	10

**Fig. 4:**
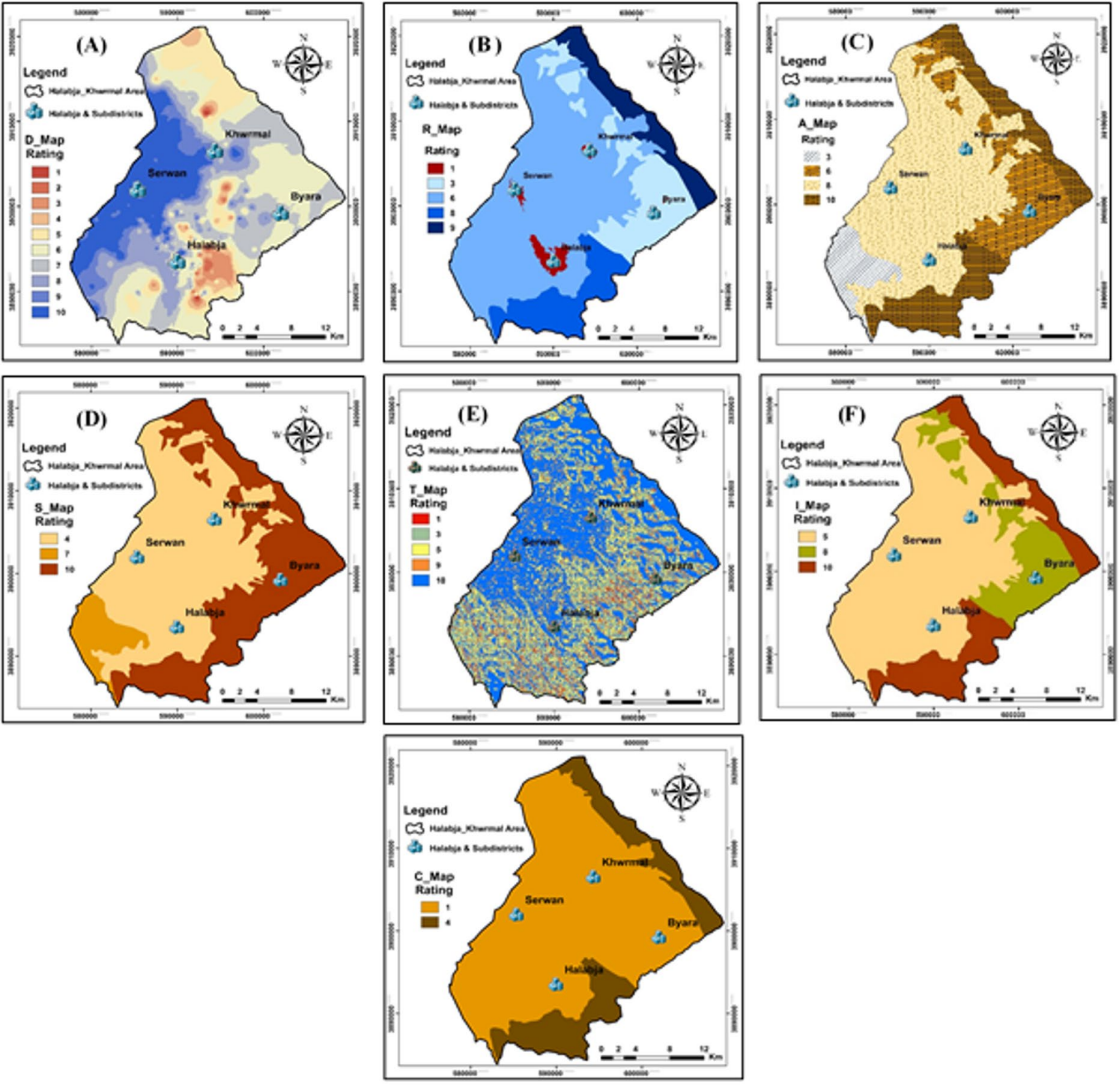
DRASTIC input layers for groundwater vulnerability mapping: **(a)** Depth to water, **(b)** Recharge, **(c)** Aquifer media, **(d)** Soil, **(e)** Slope, **(f)** Vadose zone, **(g)** Hydraulic conductivity. (This figure was prepared using ArcGIS Desktop 10.8 (https://www.esri.com/en-us/arcgis/products/arcgis-desktop/overview)).

All thematic layers were standardized to a 30 m spatial resolution prior to index calculation (Fig. 4 (a–g). The DRASTIC Index (DI) was calculated using weighted linear summation (Eq. 2):

2 $$\:DI=({D}_{r}\times\:{D}_{w})+({R}_{r}\times\:{R}_{w})+({A}_{r}\times\:{A}_{w})+({S}_{r}\times\:{S}_{w})+({T}_{r}\times\:{T}_{w})+({I}_{r}\times\:{I}_{w})+({C}_{r}\times\:{C}_{w})$$

where $\:r\:$denotes the assigned parameter rating (1–10) and $\:w\:$represents the corresponding DRASTIC weight (1–5). The resulting vulnerability index was subsequently classified into ordinal categories (low, moderate, high) to facilitate spatial interpretation and integration with groundwater potential results.

### Model validation

Model validation was performed independently for groundwater potential and groundwater vulnerability prior to their integration within the Potential–Vulnerability framework. Validation datasets were not used during model construction.

#### Groundwater potential

Groundwater potential was examined using discharge data from 430 wells, reflecting borehole productivity as an observational reference for groundwater occurrence^36^. The recorded yields were arranged into three equal-frequency classes: low (0.13–3.47 L/s; 145 wells), moderate (3.47–7.57 L/s; 142 wells), and high (7.57–17.03 L/s; 143 wells), as outlined in Table 5.

**Table 5:** Frequency distribution of observed well yields considered in ROC-based validation.

Yield Class	Yield Range (L/s)	Number of Wells	Percentage (%)
Low	0.13–3.47	145	33.7
Moderate	3.47–7.57	142	33.0
High	7.57–17.03	143	33.3

**Fig. 5:**
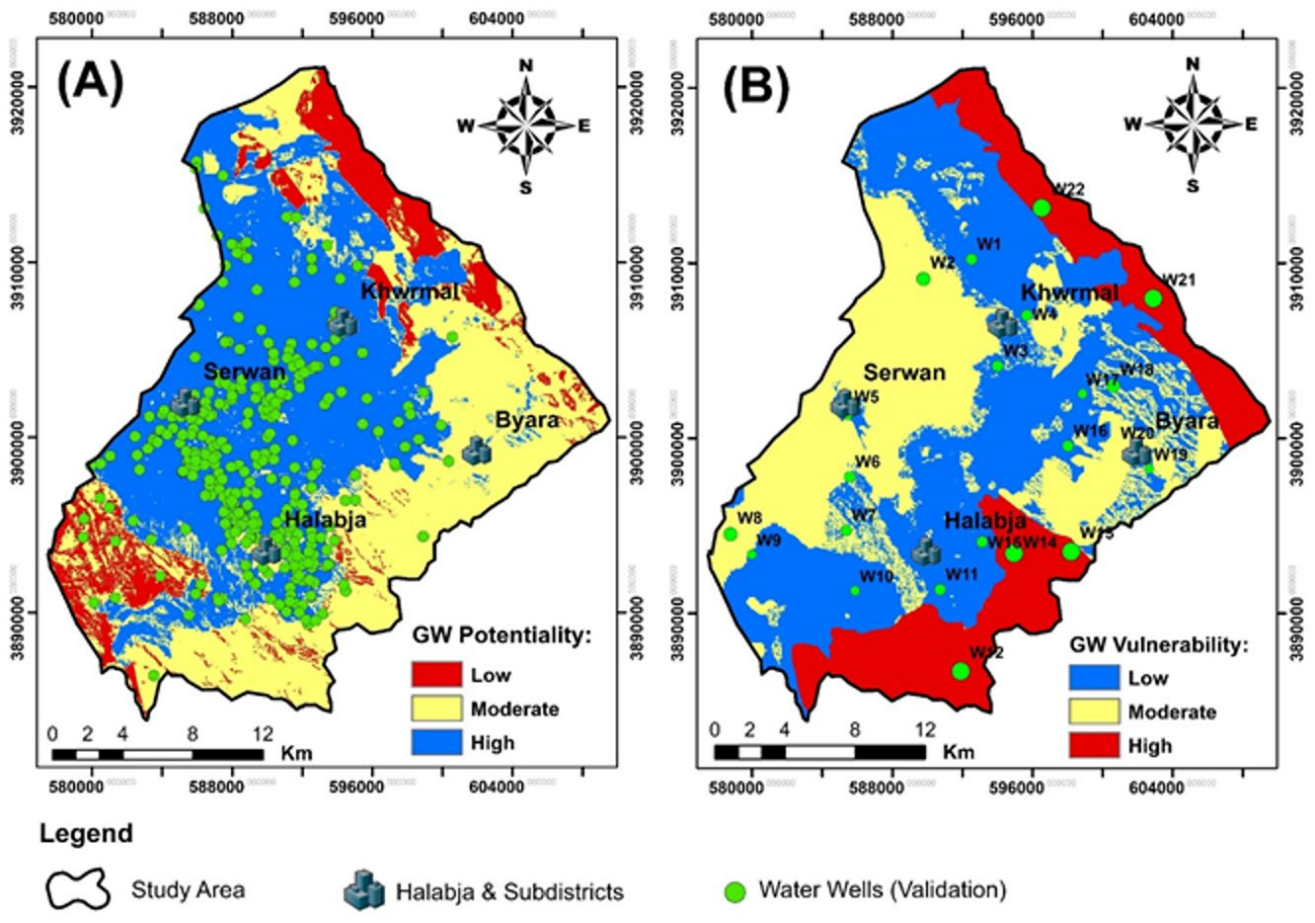
Validation outcomes: **(a)** ROC curve for GWPM; and **(b)** correlation of DI with NO₃⁻ concentrations (Fig. 5a was prepared using ArcGIS Desktop 10.8 (https://www.esri.com/en-us/arcgis/products/arcgis-desktop/overview), and Fig. 5b was prepared using Microsoft Excel 2021 (https://www.microsoft.com/microsoft-365/excel)).

For ROC analysis, wells within the high-yield class were treated as positive instances, whereas the low and moderate groups were considered as non-high-yield conditions. The resulting AUC value of 0.751 reflects a measurable separation between locations associated with elevated productivity and those characterized by lower discharge, indicating that the AHP-derived index captures the spatial expression of productive zones to a reasonable degree (Fig. 5a).

#### Groundwater vulnerability

Groundwater vulnerability was examined through comparison between the DRASTIC index and measured nitrate (NO₃⁻) concentrations, consistent with the use of nitrate as an indicator for evaluating intrinsic aquifer susceptibility^37^. Nitrate was adopted due to its mobility and its frequent association with surface-derived inputs. Regression between index values and nitrate concentrations at the 22 sampling locations, yielded a coefficient of determination of R² = 0.797, reflecting a pronounced correspondence between mapped vulnerability patterns and observed contaminant presence (Fig. 5b).

These results support the representational reliability of both models prior to their integration into the Potential–Vulnerability zonation scheme.

### Integration of groundwater potential and vulnerability

Groundwater potential and intrinsic vulnerability were integrated to produce a combined Potential–Vulnerability Index (PVI) that reflects both aquifer productivity and contamination susceptibility. Prior to integration, the continuous groundwater potential (GWPM) and DRASTIC vulnerability (GWVM) outputs were independently reclassified into three ordinal categories: low (1), moderate (2), and high (3). This reclassification preserved the internal ranking structure of each model and avoided distortion associated with direct normalization of continuous index values.

Integration was performed at the ordinal class level using a structured encoding approach (Eq. 3):

3 $$\:PV{I}_{class}=10\times\:GWP{M}_{class}+GWV{M}_{class}$$

where $\:GWP{M}_{class}\:$ and $\:GWV{M}_{class\:}$represent the ordinal class values (1–3) of groundwater potential and vulnerability, respectively.

The multiplication factor applied to groundwater potential does not represent a weighting adjustment. Instead, it serves as a positional encoding mechanism to ensure numerical separation between class combinations. By assigning groundwater potential to the tens position and vulnerability to the unit’s position, each potential–vulnerability pair produces a unique two-digit identifier (e.g., 11, 12, …, 33). This prevents class overlap and preserves the independent diagnostic meaning of both indices without rescaling or normalization. Because integration operates on ordinal classes, productivity and vulnerability remain analytically independent within the encoding scheme. High potential does not offset high vulnerability, and low potential areas remain distinct regardless of susceptibility level.

Because integration operates on ordinal classes, productivity and vulnerability remain analytically independent. High potential does not offset high vulnerability, and low potential areas remain distinct regardless of susceptibility level. The resulting PVI comprises nine discrete combinations representing all class intersections. The final map delineates zones appropriate for regulated abstraction and areas requiring protection.

## Results and discussion

### Groundwater potential and vulnerability assessment

**Fig. 6:**
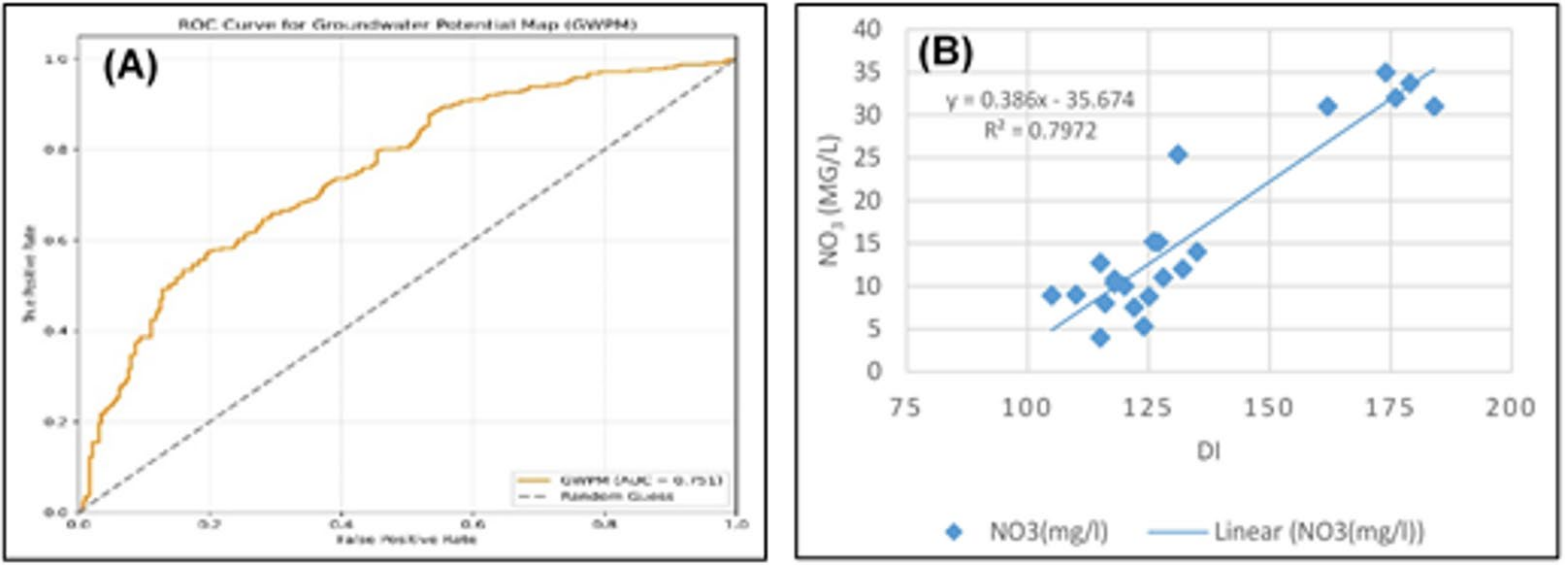
Groundwater assessment maps: **(a)** groundwater potential; **(b)** groundwater vulnerability. (This figure was prepared using ArcGIS Desktop 10.8 (https://www.esri.com/en-us/arcgis/products/arcgis-desktop/overview)).

The AHP-derived Groundwater Potential Index (GWPI) was computed in spatial form and subsequently arranged into three ordinal classes (low, moderate, and high), from which the groundwater potential map was obtained (Fig. 6a). The DRASTIC Index (DI) was treated in a similar manner and grouped into low, moderate, and high intrinsic vulnerability categories to produce the groundwater vulnerability map (Fig. 6b). The proportional extent of these classes is presented in Table 6.

**Table 6:** Spatial classification of groundwater potential and vulnerability zones.

No.	Potential Class	Area Coverage (%)	Vulnerability Class	Area Coverage (%)
1	High	49.24	High	20.65
2	Moderate	39.22	Moderate	36.15
3	Low	11.53	Low	43.18

High groundwater potential accounts for 49.24% of the study area, while moderate and low classes cover 39.22% and 11.53%. The high-potential category is concentrated mainly within carbonate units influenced by structural features and within intergranular alluvial deposits, where lithological suitability, fracture occurrence, gentle slopes, and moderate drainage conditions are observed together. In contrast, low-potential zones are found largely across steeper upland terrains and more compact formations where permeability remains restricted and recharge appears limited.

A different spatial arrangement is observed in the vulnerability distribution. Low-vulnerability zones occupy 43.18% of the basin, followed by moderate zones at 36.15% and high-vulnerability areas at 20.65%. Higher vulnerability is noted in areas associated with relatively shallow groundwater levels and more permeable vadose materials, whereas deeper groundwater conditions and less permeable formations are generally linked with lower susceptibility.

Validation outcomes correspond with these mapped patterns. The groundwater potential model yielded an Area Under the Curve (AUC) value of 0.751 from ROC analysis (Fig. 5a), reflecting separation between higher-yield and lower-yield wells. A positive relationship was also observed between DRASTIC index values and measured nitrate concentrations (R² = 0.797; Fig. 5b), where higher intrinsic vulnerability coincides with increased nitrate presence in groundwater.

### Integrated potential–vulnerability (PV) zonation

**Fig. 7:**
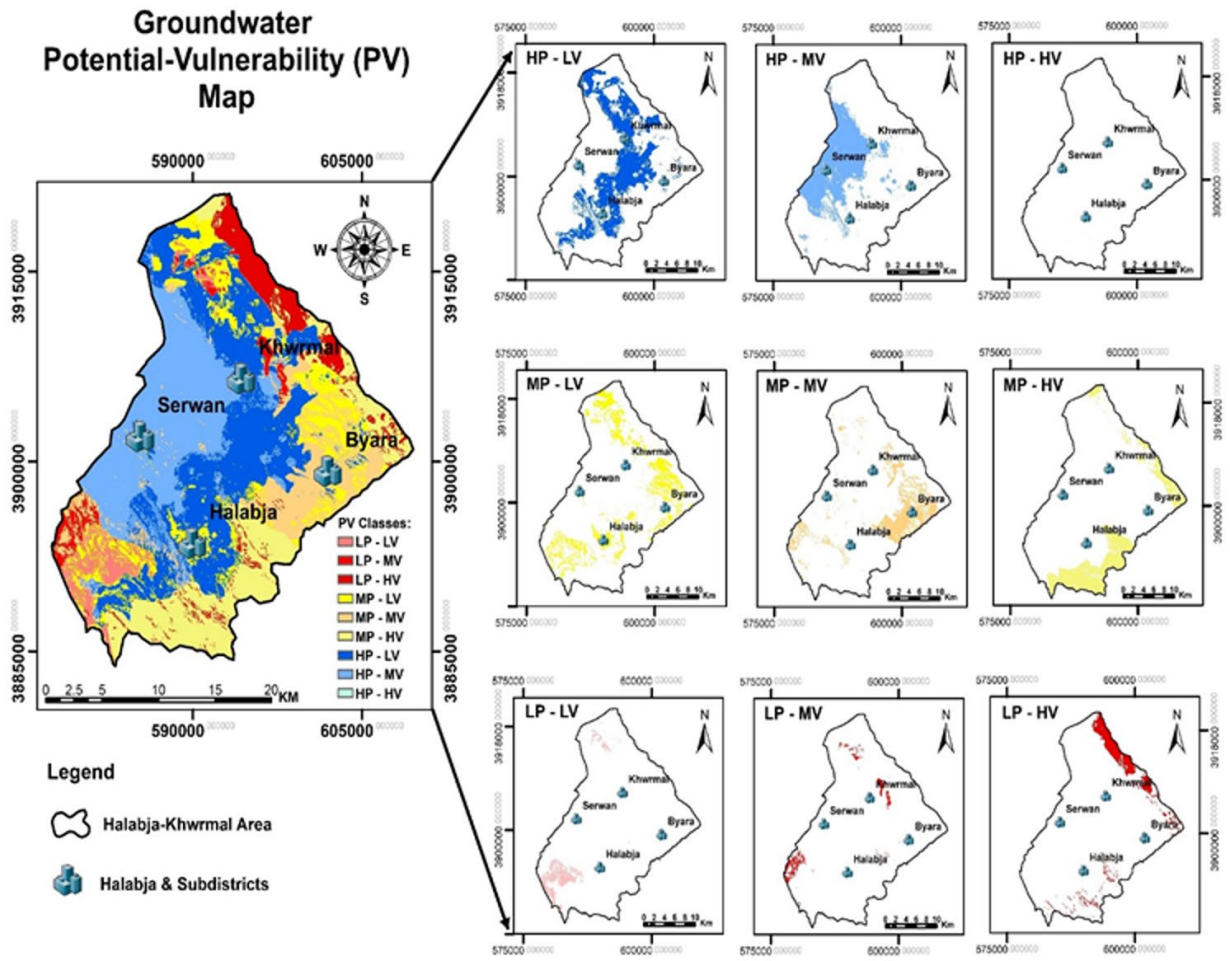
Integrated groundwater potential–vulnerability (PV) map of the study area. (This figure was prepared using ArcGIS Desktop 10.8 (https://www.esri.com/en-us/arcgis/products/arcgis-desktop/overview)).

The integration of groundwater potential and intrinsic vulnerability through the class-based Potential–Vulnerability Index (PVI) resulted in nine discrete PV classes (codes 11–33), representing all possible intersections between potential (low–moderate–high) and vulnerability (low–moderate–high). The spatial distribution and areal proportions of these combinations are summarized in Table 7, and the corresponding PV map is presented in Fig. 7.

**Table 7:** Classification and areal distribution of combined groundwater potential–vulnerability zones.

PV Matrix Code	Potential	Vulnerability	PV Class	Area (km²)	Area (%)	Management Relevance
11	Low	Low	LP–LV	21.67	3.52	Indicates limited groundwater use potential
12	Low	Moderate	LP–MV	12.67	2.06	Reflects low abstraction suitability with moderate sensitivity
13	Low	High	LP–HV	36.80	5.98	Associated with high protection sensitivity
21	Moderate	Low	MP–LV	80.29	13.05	Indicates potential for regulated groundwater use
22	Moderate	Moderate	MP–MV	71.02	11.55	Reflects balanced productivity and sensitivity
23	Moderate	High	MP–HV	90.01	14.63	Represents restricted abstraction conditions
31	High	Low	HP–LV	163.88	26.64	Indicates favorable groundwater utilization conditions
32	High	Moderate	HP–MV	138.83	22.56	Reflects development potential under sensitivity constraints
33	High	High	HP–HV	0.10	0.02	Reflects coexistence of high productivity and high vulnerability

Among these, the high potential–low vulnerability (HP–LV) class occupies the largest portion of the basin, extending over 163.88 km² (26.64%). High potential–moderate vulnerability (HP–MV) follows with 138.83 km² (22.56%). Moderate potential–high vulnerability (MP–HV) covers 90.01 km² (14.63%), while moderate potential–low vulnerability (MP–LV) accounts for 80.29 km² (13.05%). The moderate potential–moderate vulnerability (MP–MV) class spans 71.02 km² (11.55%).

Areas associated with low potential appear less extensively. Low potential–high vulnerability (LP–HV) occupies 36.80 km² (5.98%), low potential–low vulnerability (LP–LV) extends over 21.67 km² (3.52%), and low potential–moderate vulnerability (LP–MV) covers 12.67 km² (2.06%).

The high potential–high vulnerability (HP–HV) combination is limited to 0.10 km² (0.02%), indicating that the concurrence of maximum productivity and highest intrinsic vulnerability remains rare within the area.

### Discussion

Groundwater occurrence across the basin appears closely related to lithological contrasts, structural discontinuities, and terrain configuration. Fractured carbonate formations and coarse alluvial deposits, particularly where slope gradients are limited, coincide with higher productivity, whereas compact lithologies and steeper uplands tend to correspond with reduced yields. Similar structural and geomorphological controls have been documented in semi-arid aquifer systems^38,39^. The AHP-based model produced an AUC value of 0.751, indicating correspondence between the selected factors and observed well productivity patterns within the area. The role of land use in modifying recharge under favorable infiltration conditions has also been reported in comparable settings^40^.

Intrinsic vulnerability shows a predominantly low to moderate character. Deeper groundwater levels and less transmissive vadose materials provide greater attenuation potential across much of the basin. Elevated vulnerability remains spatially restricted and is generally associated with shallow groundwater and permeable unsaturated materials, conditions commonly identified in vulnerability assessments of semi-arid basins^41,42^. The correspondence between DRASTIC index values and measured nitrate concentrations (R² = 0.797) indicates that areas classified as more vulnerable tend to coincide with higher contamination levels, consistent with findings reported by^11^. Although nitrate distribution reflects land-use intensity, its spatial alignment with intrinsic vulnerability points to the influence of hydrogeological structure on contaminant migration.

When groundwater potential and vulnerability are considered jointly, their spatial patterns do not appear uniformly aligned. High potential–low vulnerability areas occupy the largest portion of the basin, while zones characterized simultaneously by high productivity and high intrinsic susceptibility are nearly absent. This limited spatial coincidence indicates that transmissive formations enhancing productivity are not systematically associated with reduced natural protection. The integrated framework therefore distinguishes between resource availability and intrinsic sensitivity rather than assuming their overlap.

#### Methodological considerations

The framework retains inherent constraints. AHP weighting involves expert judgment, even where consistency metrics are satisfied. The DRASTIC model represents intrinsic susceptibility and does not incorporate temporal variability, pumping-induced flow alteration, or contaminant-specific transport dynamics, a limitation increasingly recognized in vulnerability assessments that distinguish between static and dynamic influences on aquifer sensitivity^43^. Validation relied on nitrate as a representative indicator; inclusion of additional hydrochemical tracers could refine interpretation. Nevertheless, validation of both indices prior to integration strengthens confidence in the resulting PV zonation.

## Conclusion

This study presents an integrated Potential–Vulnerability (PV) framework that combines AHP-based groundwater potential mapping with intrinsic vulnerability assessment using the standard DRASTIC model in the Halabja–Khwrmal basin. Independent validation showed acceptable discrimination of groundwater productivity (AUC = 0.751) and a strong spatial association between vulnerability index values and measured nitrate concentrations (R² = 0.797), supporting the reliability of both component models.

The integrated zonation reveals a non-uniform relationship between groundwater availability and intrinsic susceptibility. High potential–low vulnerability and high potential–moderate vulnerability classes together occupy a substantial portion of the basin, where favorable aquifer conditions occur under comparatively moderate intrinsic risk. In contrast, zones combining elevated vulnerability with moderate or low productivity delineate areas where hydrogeological sensitivity may constrain resource use. The very limited extent of the high potential–high vulnerability class indicates minimal spatial coincidence between maximum productivity and highest intrinsic susceptibility.

Rather than relying on numerical compensation between indices, the PV framework preserves ordinal class relationships, allowing groundwater productivity and intrinsic sensitivity to be evaluated jointly without masking either condition. Although the approach remains influenced by expert-based weighting, the static nature of intrinsic vulnerability assessment, and reliance on nitrate for validation, the resulting zonation provides a spatial basis for groundwater planning in semi-arid environments where resource availability and contamination susceptibility must be considered simultaneously. The framework may be transferable to structurally variable semi-arid basins facing similar groundwater management constraints.

## Data Availability

Data sets not publicly available can be obtained from the corresponding author upon reasonable request.
